# Root Bark of *Paeonia suffruticosa* Extract and Its Component Methyl Gallate Possess Peroxynitrite Scavenging Activity and Anti-Inflammatory Properties through NF-κB Inhibition in LPS-treated Mice

**DOI:** 10.3390/molecules24193483

**Published:** 2019-09-25

**Authors:** Dong Jin Park, Hee Jin Jung, Chan Hum Park, Takako Yokozawa, Ji-Cheon Jeong

**Affiliations:** 1 Department of Internal Medicine, College of Korean Medicine, Dongguk University, 27, Dongguk-ro, Ilsandong-gu, Goyang-si, Gyeonggi-do 10326, Korea; 26spirit@daum.net; 2 Longevity Life Science and Technology Institutes, Pusan National University, Busan 46241, Korea; king2046@daum.net; 3 Department of Medicinal Crop Research, National Institute of Horticultural and Herbal Science, Rural Development Administration, Eumseong 369-873, Korea; ptman123@korea.kr; 4 Graduate School of Science and Engineering for Research, University of Toyama, Toyama 930-8555, Japan

**Keywords:** peroxynitrite, medicinal plant, *Paeoniasuffruticosa* Andrew, methyl gallate, lipopolysaccharide, anti-inflammatory activity

## Abstract

A peroxynitrite (ONOO^−^)-generating system induced by 3-morpholinosydnonimine, was used to evaluate the ONOO^−^ scavenging properties of plants that have been widely used as traditional medicine in Korea for the treatment of several diseases. The most effective medicinal plants were *Paeonia suffruticosa* Andrew, followed in order by *Lonicera japonica* Thunb., *Curcuma zedoaria* (Christm.) Roscoe, and *Pueraria thunbergiana* Benth. In addition, root bark of *P. suffruticosa* was partitioned with organic solvents of different polarities, and the ethyl acetate (EtOAc) fraction showed the strongest ONOO^−^ scavenging activity. Methyl gallate, a plant-derived phenolic compound identified from the EtOAc fraction, exerted strong ONOO^−^ scavenging activity. The in vivo therapeutic potential of methyl gallate was investigated using lipopolysaccharide-treated mice. Oral administration of methyl gallate protected against acute renal injury and exhibited potential anti-inflammatory properties through an increase in antioxidant activity and decrease in nuclear factor-kappa B activity.

## 1. Introduction

It is widely recognized that oxidative stress caused by excessive reactive species (RS) including reactive oxygen species (ROS) and reactive nitrogen species (RNS) is associated with the pathogenesis of diseases [1]. Peroxynitrite (ONOO^−^), a powerful oxidant formed through the reaction of nitric oxide (NO) and superoxide (O_2_^−^), is thought to be cytotoxic itself and is capable of breaking down into other toxic species, such as the hydroxyl radical (·OH) [2]. RNS are also known to be active nuclear factor-kappa B (NF-κB), redox sensitive, and pro-inflammatory transcription factor [3]. Antioxidants that protect against oxidative damage induced by free radicals, prevent the onset and progression of disease [4,5]. Therefore, research interests are focused on the development of safe, effective, and non-toxic antioxidants.

From ancient times, humans have benefited from natural plants and compounds. It has been generally recognized that traditional oriental medicines plays a unique role in the prevention and treatment of many human diseases associated with excess free radicals. Koreans have also used and cultivated a variety of plants with medicinal function, which are speculated to be potent ONOO^−^ scavengers that can protect tissues and cells from damage caused by excess RS. ONOO^−^ and ROS scavengers can provide promising indicators of new treatments.

Currently, several methods and procedures, have been established to evaluate radical scavenging and antioxidant activity both in vivo and in vitro [6,7,8,9]. In this study, we used a 3-morpholinosydnonimine (SIN-1)-induced ONOO^−^-generating system, because it can be used to assay a large number of samples within a short period and is sensitive enough to detect natural antioxidants at low concentrations. We used this system to conduct the primary screening for the presence of radical scavenging activity among various medicinal plants, their fractions, and active compounds. We also investigated the effects of methyl gallate, a plant-derived phenolic compound found in root bark of *Paeonia suffruticosa* Andrew, on oxidative stress and inflammation in lipopolysaccharide (LPS)-treated mice.

## 2. Results

### 2.1. ONOO^−^ Scavenging Activity of 70% EtOH Extracts from Korean Medicinal Plants

To evaluate the oxidative status, 70% EtOH extracts from seven different medicinal plants (Table 1) were screened using a dihydrorhodamine 123 (DHR 123)probe (Table 2). As shown in Table 2, the most potent ONOO^−^ scavenger was *Paeonia* *suffruticosa* Andrew, which showed an IC_50_ value of 7.69 µg/mL. *Lonicera japonica* Thunb. also showed a strong scavenging effect, with an IC_50_ value below 8 µg/mL. In addition, *Curcuma zedoaria* (Christm.) Roscoe, *Pueraria thunbergiana* Benth, and *Scirpus yagara* Ohwi were effective ONOO^−^ scavengers with IC_50_ values of 10.13, 10.65 and 11.74 µg/mL, respectively. All the plants tested had IC_50_ values below 14 µg/mL.

**Figure 1:**
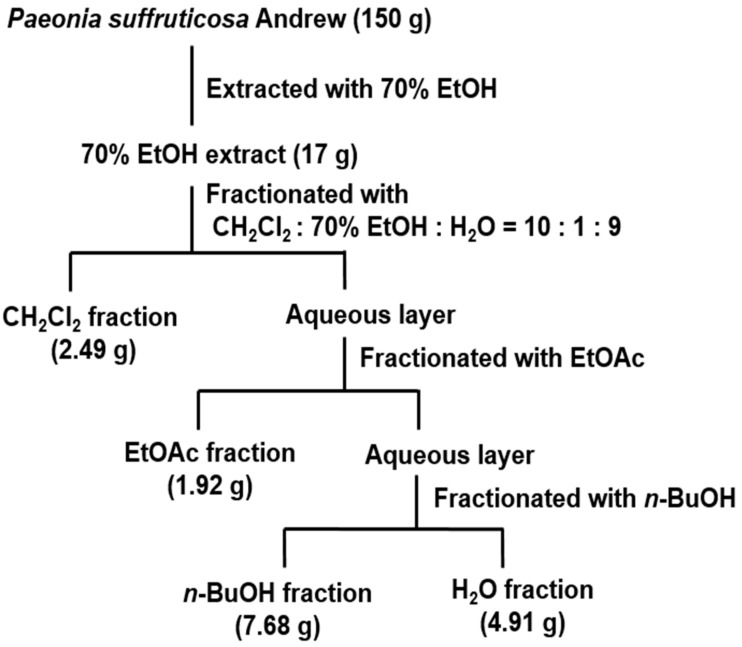
Extraction and fractionation from root bark of *P. suffruticosa*.

After separation of the root bark of *P.* *suffruticosa* extracts, which is the strongest ONOO^−^ scavenger, a difference in scavenging activity was observed among the various solvent-soluble fractions (Figure 1). As shown in Table 3, the ONOO^−^ scavenging activity of dichloromethane (CH_2_Cl_2_) and *n*-butanol (*n*-BuOH) fractions were stronger than that exhibited by the 70% EtOH extract. Moreover, the ethyl acetate (EtOAc) fraction showed the strongest, ONOO^−^ scavenging activity with an IC_50_ value of 0.25 µg/mL.

**Figure 2:**
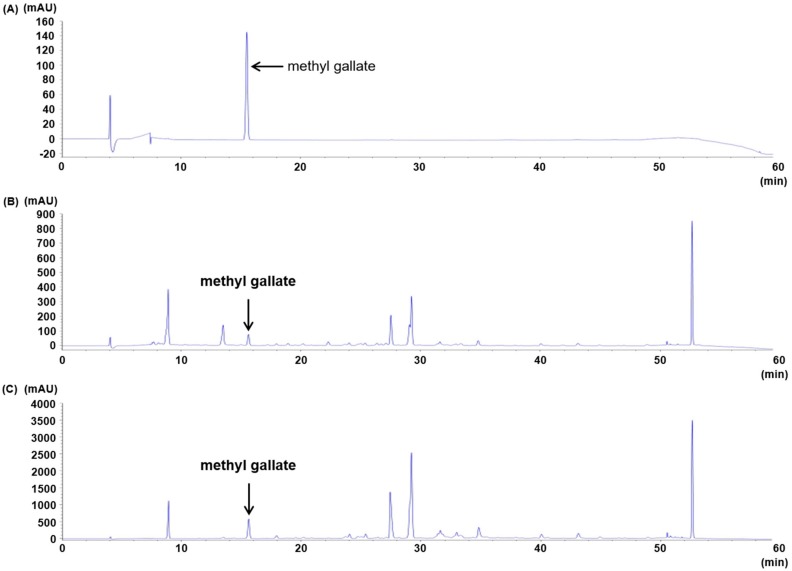
HPLC chromatograms of methyl gallate (**A**), 70% EtOH extract from *P. suffruticosa* (**B**), and EtOAc fraction (**C**) of the 70% EtOH extract.

Methyl gallate was detected in the EtOAc fraction from root bark of *P.* *suffruticosa* extracts (Figure 2, Table 4 and Table 5) and exhibited a high ONOO^−^ scavenging activity with an IC_50_ of 0.91 µM compared to that of the positive control l-penicillamine with an IC_50_ value of 8.79 µM (Table 6). 

### 2.2. Effect of Root Bark of P. suffruticosa Extracts and Methyl Gallate on LPS-Induced Kidney Injury in Mice

**Figure 3:**
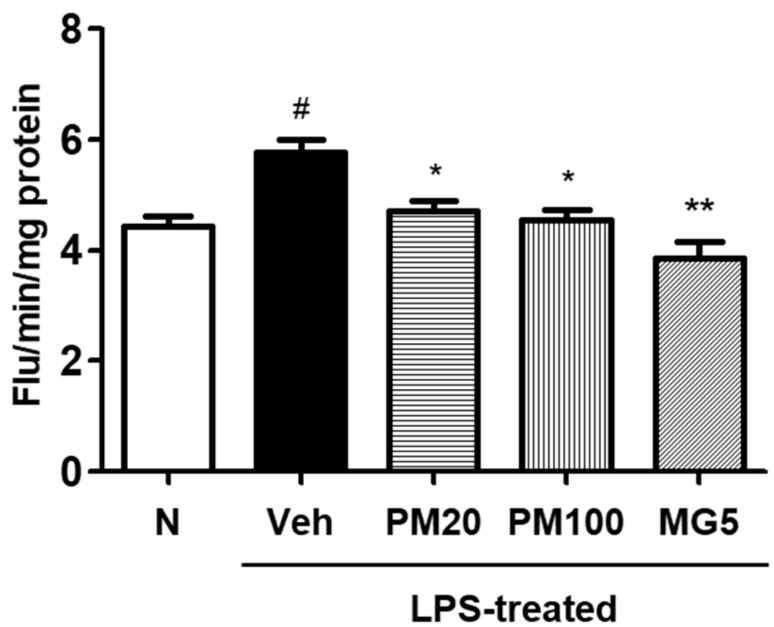
ROS content in the kidney. N, normal mice, Veh, vehicle-administered and LPS-treated mice; PM20, *P. suffruticosa* 20 mg/kg body weight-administered and LPS-treated mice; PM100, *P. suffruticosa* 100 mg/kg body weight-administered and LPS-treated mice; and MG5, methyl gallate 5 mg/kg body weight-administered and LPS-treated mice. One-factor ANOVA: # *p* < 0.05 versus normal mice; * *p* < 0.05 and ** *p* < 0.01 versus veh (vehicle-administered and LPS-treated mice). Bars indicate standard errors of means (S.E.M.).

ROS levels in the kidneys of LPS-treated mice were higher than that of normal mice (Figure 3). However, ROS production was significantly inhibited by treatment with both root bark of *P.* *suffruticosa* extract (20 or 100 mg/kg body weight) and methyl gallate (5 mg/kg body weight) (Figure 3).

**Figure 4:**
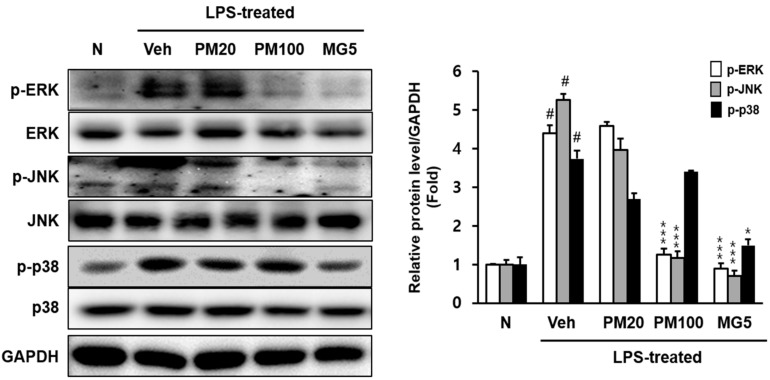
Total and phosphorylated p-ERK, p-JNK, and p-p38 protein levels in the kidney. N, normal mice; Veh, vehicle-administered and LPS-treated mice; PM20, *P. suffruticosa* 20 mg/kg body weight-administered and LPS-treated mice; PM100, *P. suffruticosa* 100 mg/kg body weight-administered and LPS-treated mice; and MG5, methyl gallate 5 mg/kg body weight-administered and LPS-treated mice. The protein levels of p-ERK, p-JNK, and p-p38 were quantified using CS analyzer software. Respective total-form proteins were used as loading control. A representation of three experiments that yielded similar results. One-factor ANOVA: # *p* < 0.05 versus normal mice; * *p* < 0.05 and *** *p* < 0.001 versus veh (vehicle-administered and LPS-treated mice). Bars indicate standard errors of means (S.E.M.).

Phosphorylated extracellular signal-regulated kinase (p-ERK), phosphorylated c-Jun N-terminal kinase (p-JNK), and phosphorylated p-38 (p-p38) protein levels increased in the kidneys of LPS-treated mice (Figure 4). 

The oral administration of 100 mg/kg root bark of *P.* *suffruticosa* extract and 5 mg/kg methyl gallate significantly reduced the expression of these two mitogen-activated protein kinase (MAPK)-related proteins (p-ERK and p-JNK). The level of p-p38 significantly decreased in response to treatment with 5 mg/kg methyl gallate administration (Figure 4).

**Figure 5:**
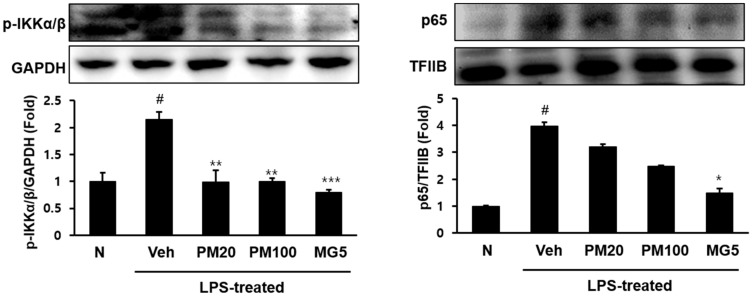
Phosphorylated IKKα/β and p65 protein levels in the kidney. N, normal mice; Veh, vehicle-administered and LPS-treated mice; PM20, *P. suffruticosa* 20 mg/kg body weight-administered and LPS-treated mice; PM100, *P. suffruticosa* 100 mg/kg body weight-administered and LPS-treated mice; and MG5, methyl gallate 5 mg/kg body weight-administered and LPS-treated mice. The protein levels of IKKα/β and p65 were quantified using CS analyzer software. GAPDH and transcription factor II B (TFIIB) were used as loading control in cytosolic and nucleus fraction, respectively. A representation of three experiments that yielded similar results. One-factor ANOVA: # *p* < 0.05 versus normal mice; * *p* < 0.05, ** *p* < 0.01 and *** *p* < 0.001 versus veh (vehicle-administered and LPS-treated mice). Bars indicate standard errors of means (S.E.M.).

The phosphorylation levels of the NF-κB-related protein inhibitor-κB kinase inhibitor of nuclear kappa-B kinase (IKK) α/β, and nuclear p65 increased in the kidney in response to LPS treatment; however, methyl gallate-treated mice expressed significantly lower levels of these proteins relative to the normal mice. Treatment with 100 mg/kg root bark of *P.* *suffruticosa* extract also decreased the LPS-induced increase in IKKα/β and nuclear p65 (Figure 5).

**Figure 6:**
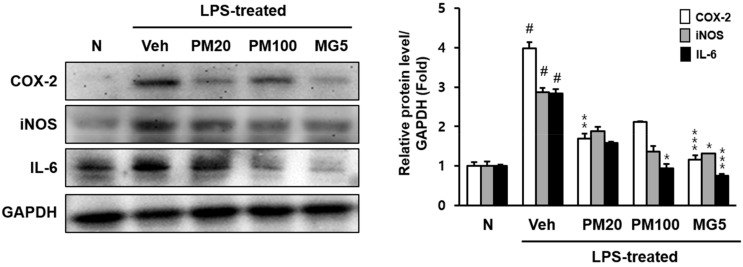
COX-2, iNOS, and IL-6 protein levels in the kidney. N, normal mice; Veh, vehicle-administered and LPS-treated mice; PM20, *P. suffruticosa* 20 mg/kg body weight-administered and LPS-treated mice; PM100, *P. suffruticosa* 100 mg/kg body weight-administered and LPS-treated mice; and MG5, methyl gallate 5 mg/kg body weight-administered and LPS-treated mice. The protein levels of COX-2, iNOS, and IL-6 were quantified using CS analyzer software. GAPDH was used as loading control. A representation of three experiments that yielded similar results. One-factor ANOVA: # *p* < 0.05 versus normal mice; * *p* < 0.05, ** *p* < 0.01 and *** *p* < 0.001 versus veh (vehicle-administered and LPS-treated mice). Bars indicate standard errors of means (S.E.M.).

We quantified renal cyclooxygenase-2 (COX-2), inducible nitric oxide synthase (iNOS), and interleukin (IL)-6 protein expression levels, whose activations are involved in ROS and related to inflammatory responses (Figure 6). COX-2, iNOS, and IL-6 protein expression in LPS-treated mice significantly increased compared to those observed in normal mice; however, all three proteins were downregulated in response to methyl gallate treatment. Specially, COX-2 and IL-6 protein expression methyl gallate-treated mice reduced nearly to the levels observed in normal mice or lower (Figure 6).

## 3. Discussion

Antioxidants that protect against oxidative damage induced by free radicals, prevent the onset and progression of diseases. Research interests have been focused on the development of safe, effective, and non-toxic antioxidants. Medicinal plants are also thought to be potent free radical scavengers that can protect tissues and cells from injury caused by exposure to excess free radicals and may provide novel therapies for various pathological conditions. Therefore, we investigated the ONOO^−^ scavenging properties of medicinal plants.

Among the medicinal plants studied, root bark of *P.* *suffruticosa* exhibited strong ONOO^−^ scavenging activity. It has been used extensively in Korea and other countries in eastern Asian, as traditional medicine for the treatment of various diseases. A variety of compounds, such as oxypaeonifloin, paeoniflorin, albiflorin, benzoyl albiflorin, gallic acid, pentagalloylglucose, paeonol and benzoic acid make up its bioactive constituents [10,11]. These polyphenolic compounds have been reported to improve memory [12], possess antioxidant capabilities [13], confer hepatoprotection [14], and possess anti-atherosclerotic [15], antimutagenic [16], and antiplatelet aggregation properties [17].

The 70% EtOH extract of from root bark of *P.* *suffruticosa* has been shown to exhibit an ONOO^−^ scavenging effect and was therefore partitioned with organic solvents of different polarities to afford CH_2_Cl_2_, EtOAc, *n*-BuOH fractions, and H_2_O residue (Figure 1). The EtOAc fraction exhibited the strongest ONOO^−^ scavenging activity, with an IC_50_ value of 0.25 μg/mL. Then the EtOAc fraction was subjected to reverse-phase high-performance liquid chromatography (HPLC), and methyl gallate was identified (Figure 2). This compound showed strong ONOO^−^ scavenging properties with an IC_50_ value of 0.91 μM, whereas the derivatives of methyl gallate such as ethyl gallate, propyl gallate, and butyl gallate showed weak ONOO^−^ scavenging (data not shown). Therefore, we investigated the antioxidant effect of methyl gallate in vivo, in an animal model of oxidative stress.

Many studies have demonstrated that endotoxemia, sepsis, and septic shock are associated with the generation of ROS. LPS promotes production of ROS, such as O_2_^−^, hydrogen peroxide, and ·OH in macrophages [18]. After LPS administration, the NO produced is a potent inflammatory mediator that reacts with O_2_^−^ and produces ONOO^-^ [19,20,21,22]. Therefore, this study evaluated the effect of methyl gallate on LPS-treated renal injury in mice.

Oxidative stress plays an important role in pathological conditions of the kidney. We have demonstrated that increased oxidative stress may significantly contribute to the development of renal conditions that lead to structural and functional changes, similar to those observed in the LPS-treated model [23]. In this study, mice with LPS-treated renal injury were found to be more susceptible to oxidative damage; however, methyl gallate treatment suppressed renal ROS levels, suggesting that methyl gallate protected oxidative stress damage of kidneys in mice (Figure 3).

Increased oxidative stress-related MAPKs (p-ERK, p-JNK, and p-p38) protein expressions was also observed in the renal tissues of the LPS-treated mice and decreased with the administration of methyl gallate (Figure 4). The MAPKs pathway is activated by oxidative stress, resulting in inflammation, apoptosis, and the development of renal lesions in mice with LPS-induced renal injury [24]. Conversely, methyl gallate treatment in these LPS-treated mice reduced p-ERK, p-JNK, and p-p38 activation. The results suggest that methyl gallate has a crucial effect on oxidative stress, and oxidative stress mediates the activation of the MAPKs-related NF-κB signaling pathway in renal tissue.

NF-κB is a major pro-inflammatory transcription factor that plays a key role in the regulation of transcription and the expression of many genes producing a wide range of pro-inflammatory molecules including cytokines, chemokines, adhesion molecules, and inflammatory enzymes, such as COX-2 and iNOS [25]. Upon activation by LPS, inhibitor of kappa B (IκB) α is rapidly phosphorylated and degraded, leading to the release of NF-κBp65. After which, NF-κB translocates to the nucleus and promotes the transcription of target genes such as tumor necrosis factor α, IL-1, and IL-6 [26]. Increased IL-6 protein expression is significantly lowered with the administration of methyl gallate (Figure 6).

Furthermore, LPS is a well-known as a potent stimulator of iNOS expression, leading to the overproduction of NO [27]. The iNOS-induced excessive NO plays a key role by directly inducing tissue dysfunction and formation of ONOO^−^. Inhibition of iNOS is critical for the alleviation of acute renal injury. We demonstrated that the protein levels of renal iNOS, which generates NO and COX-2, and in turn generates prostaglandin E_2_ and ROS, significantly improved in LPS-treated mice. In contrast, methyl gallate administration significantly suppressed these proteins.

**Figure 7:**
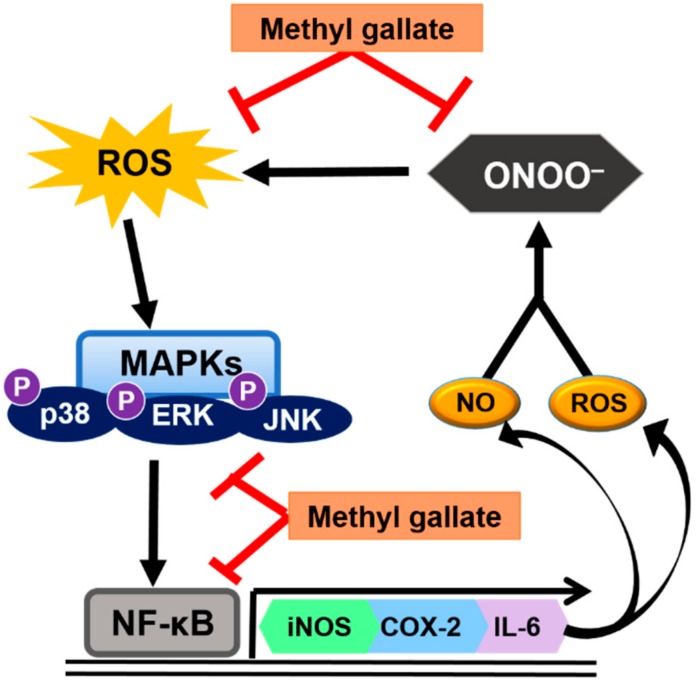
Predicted activity of methyl gallate in renal tissue.

As summarized in Figure 7, methyl gallate is protective against acute kidney injury and exhibits strong anti-inflammatory properties through increased antioxidant activity and reduce IκBα degradation and NF-κB activity. This study suggests that methyl gallate may exert its kidney-protecting potential by inhibiting the oxidative stress-sensitive mechanisms of the proinflammatory response. Methyl gallate is a plant-derived phenolic compound found in various plants and natural products including *Schinus terebinthifolius*, *Rosa rugose*, and *Galla rhois* [28,29,30]. It has been extensively studied because of its antioxidant, antitumor, and antimicrobial activities [31,32,33]. In relation to its antioxidant effects, methyl gallate has been shown to be a free radical scavenger that inhibits lipid peroxidation and protects against DNA damage due to oxidative stress [34,35]. Our current results suggest that methyl gallate may be an important factor in the prevention of kidney disorders caused by LPS.

## 4. Materials and Methods

### 4.1. Materials

LPS from *Escherichia coli* (serotype 0111:B4), diethylenetriamine pentaacetic acid (DTPA), DHR123, 3-morpholinosydnonimine (SIN-1), l-2-amino-3-mercapto-3-methylbutanoic acid (l-penicillamine), and methyl gallate were obtained from Sigma Chemical Co. (St. Louis, MO, USA). DCFH-DA was obtained from Molecular Probes (Eugene, OR, USA), and immobilon-P transfer membranes were purchased from Millipore (Bedford, MA, USA). The antibodies used were sourced as follow: The antibodies to p-ERK, ERK, p-JNK, JNK, p-p38, p38, COX-2, iNOS, IL-6, β-actin, and horseradish peroxidase (HRP)-conjugated secondary antibodies were from Santa Cruz Biotechnology (Santa Cruz, CA, USA); and p-IKKα/β and p65 were from Cell Signaling Biotechnology (Beverly, MA, USA). Enhanced chemiluminescence western blot detection reagents were purchased from Amersham Life Science (Arlington Heights, IL, USA). All chemicals and solvents used were purchased from E. Merck (Frankfurt Str., Darmstadt, Germany), Fluka (St. Louis, Mo, USA) and Sigma-Aldrich Co., unless stated otherwise.

### 4.2. Korean Medicinal Plants

As shown in Table 1, the following 7 kinds of Korean medicinal plants were prepared: *Lonicera japonica* Thunb. (whole), *Lysimachia christinae* Hance (whole), *Scirpus yagara* Ohwi. (root), *P.* *suffruticosa* Andrew (root bark), *Ailanthus altissima* (Mill.) Swingle (leaf), *Pueraria thunbergiana* Benth. (root), and *Curcuma zedoaria* (Christm.) Roscoe (root). A voucher for the herbarium specimen, identified by a plant systematist (PUREMIND R&D center), was supplied by PUREMIND Co. (Youngchunsi, Kyungpook, Korea). A voucher specimen (no. 20180911~20180917) has been deposited in the laboratory of Prof. J. C. Jeong.

### 4.3. Preparation of Extracts

The dried parts of the Korean medicinal plants were pulverized and ultrasonically extracted twice in 70% EtOH (60 Hz, room temperature, 12 h). Subsequently, the solvents were evaporated in vacuo to obtain an extract with the yield of 8–15% by weight of each original material.

### 4.4. Fractionation of the 70% EtOH Extract of Root Bark of P. suffruticosa

The 70% EtOH extract was partitioned using organic solvents of different polarities to afford CH_2_Cl_2_, EtOAc, *n*-BuOH fractions, and H_2_O residues, in sequence, as shown in Figure 1.

### 4.5. HPLC Analysis of the EtOAc Fraction of Root Bark of P. suffruticosa

The EtOAc fraction was dissolved in 10 mL of 50% MeOH with multi vortexing, and filtered through a Dismic-13 JP membrane filter (Advantec Toyo, Tokyo, Japan; pore diameter: 0.2 µm). We injected 10 µL of the sample into a reverse-phase HPLC using a YMC pack ODS-AM (4.6 × 250 mm, 5 µm pore size), with a column temperature of 35 °C. Mobile phase: A = 0.5% formic acid (aq.), B = acetonitrile. The gradient conditions were as follows: 0 min, 0% B; 4 min, 12% B; 20 min, 18% B; 24 min, 22% B; 34 min, 26% B; 44 min, 30% B; 54 min, 100% B. The flow rate was 0.8 mL/min. The UV absorbance at 254 nm was monitored using an Agilent 1200 series diode array detector (Agilent Technologies, Waldbronn, Germany). The peak was assigned by carrying out co-injection tests with an authentic sample and comparing with the UV spectral data. The measurement was repeated three times for each sample. Representative HPLC results are illustrated in Figure 2. Quantification of methyl gallate was conducted by peak area measurement. The calibration curve of the standard compound (methyl gallate) was made over a range of 1–8 µg/mL. The detector response was linear over the concentration range used. The regression coefficient (*r*^2^) of standard compound was higher than 0.996 (Table 4). The amount of methyl gallate is shown in Table 5.

### 4.6. Assay of ONOO^−^ Levels

ONOO^−^-dependent oxidation of DHR 123 to rhodamine 123 was assayed, according to the method described by Kooy et al. [36]. Samples were added to the rhodamine buffer (pH 7.4) containing 6.25 μM DHR 123 and 125 μM DTPA and incubated for 5 min at 37 °C. The absorbance was determined at 500 nm, the absorbance of rhodamine 123. The antioxidant activity of each sample was expressed in terms of the IC_50_ value (concentration in μg/mL or μM required to inhibit ONOO^−^ formation by 50%) determined from the log dose-inhibition curve. L-Penicillamine was used as positive control.

### 4.7. Experimental Animals and Treatment

All animal experiments were performed according to the Guidelines for Care and Use of Laboratory Animals (PNU-IACUC; Approval No. PNU-2018-2054). Six-week-old male C57BL/6 mice were purchased from Samtako (Osan, Korea) and housed under a 12 h light/dark cycle in a room with controlled temperature (24 °C) and humidity (55 ± 5%). After acclimatization (1 week), the mice were divided into five groups (n = 6, each), avoiding any intergroup differences in body weight. The normal and vehicle-treated LPS groups were provided water, whereas the other groups were orally administered *P. suffruticosa* at a dose of 20 or 100 mg/kg body weight, and methyl gallate at a dose of 5 mg/kg body weight daily, using a stomach tube for three consecutive days. The doses administered and the duration of treatment were determined based on the data obtained in a preliminary study. After the three day administration period, all mice except those in the normal group, were administered intraperitoneal LPS at 5 mg/kg body weight. At 5 h after LPS challenge, blood samples were collected from anesthetized mice. Subsequently, the kidney was rinsed with ice-cold physiological saline (0.9% NaCl, pH 7.4), removed, flash frozen, and stored at −80 °C until analysis.

### 4.8. Measurement of ROS Levels in the Kidney

ROS was measured according to the method described by Ali et al. [37]. Renal tissue was homogenized on ice with 1 mM ethylenediaminetetraacetic acid (EDTA)-50 mM sodium phosphate buffer (pH 7.4), and 25 mM DCFH-DA was added to the homogenate. After incubation for 30 min, changes in fluorescence values were determined at an excitation wavelength of 480 nm and emission wavelength of 535 nm.

### 4.9. Preparation of Cytosol and Nuclear Fractions

Following the method proposed by Deng et al. [38], renal tissue was homogenized with an ice-cold lysis buffer containing 10 mM Tris (pH 8.0), 20 mM NaF, 2 mM sodium orthovanadate, 20 mM β-glycerophosphate, 0.01 mM dithiothreitol (DTT), 1 mM EDTA, 0.5 mM phenylmethylsulfonyl fluoride (PMSF), 0.1% Nonidet P-40, and protease inhibitors. It was then kept on ice for 20 min and centrifuged at 12,000 rpm at 4 °C for 10 min. The supernatants were used as the cytosolic fraction. The pellets were washed three times with cytosol lysis buffer. To extract the nuclear fraction, the pellet was homogenized and suspended in 10 mM Tris (pH 8.0), 100 mM NaCl, 50 mM KCl, 10% (*v*/*v*) glycerol, 0.01 mM DTT, 0.1 mM EDTA, 20 mM β-glycerophosphate, 2 mM sodium orthovanadate, 20 mM NaF, 0.5 mM PMSF, and protease inhibitors. The suspension was incubated on ice for 30 min, and then centrifuged at 12,000 rpm for 10 min. The resultant supernatants were used as the nuclear fraction. The protein concentration in each fraction was determined using the bicinchonic acid assay method with bovine serum albumin as a standard.

### 4.10. Western Blotting

Western blotting was conducted as described previously [39]. Lysed samples were measured using bicinchoninic acid (Thermo Scientific, Waltham, MA, USA) and bovine serum albumin was used as the standard for protein concentrations. Samples were prepared in a gel buffer [12.5 mM Tris buffer (pH 6.8), 4% sodium dodecyl sulfate (SDS), 20% glycerol, 10% 2-mercaptoethanol, and 0.2% bromophenol blue] and kept at 100 °C for 5 min. SDS-polyacrylamide gel electrophoresis containing with 8% to 14% acrylamide was used to separate equal concentrations of protein. Using a wet transfer system, the gels were transferred to polyvinylidene difluoride membranes at 90 V for 90 min. Membranes were immediately incubated in blocking buffer [10 mM Tris buffer (pH 7.5), 100 mM NaCl, 0.1% Tween 20, and 5% non-fat milk]. Blotting was done at 25 °C for 30 min and then membranes were incubated with specific primary antibody at 4 °C for 16 h. The secondary antibody was then added followed by an HRP conjugated anti-rabbit, anti-goat antibody, or anti-mouse antibody at 25 °C for 1 h. Antibody labeling was used to detect antibodies with WesternBright^TM^ ECL reagent (Advansta, Menlo Park, CA, USA).

### 4.11. Statistical Analyses

All statistical analyses were performed using Graphpad Prism5 software. Analysis of variance (ANOVA) was used to analyze differences between each group, and Dunnett’s multiple comparison tests were used to determine the differences between the mean values of groups. Statistical significance was accepted for *p* values < 0.05. For western blotting, blots are representative of at least three independent experiments.

## Figures and Tables

**Table 1:** Korean medicinal plants.

Herb Name	Family	Used Part
*Lonicera japonica* Thunb.	Caprifoliaceae	Whole
*Lysimachia christinae* Hance	Primulaceae	Whole
*Scirpus yagara* Ohwi	Cyperaceae	Root
*Paeonia suffruticosa* Andrew	Paeoniaceae	Root bark
*Ailanthus altissima* (Mill.) Swingle	Simaroubaceae	Leaf
*Pueraria thunbergiana* Benth	Leguminosae/Fabaceae	Root
*Curcuma zedoaria* (Christm.) Roscoe	Zingiberaceae	Root

**Table 2:** ONOO^−^ scavenging activity of 70% EtOH extract from Korean medicinal plants.

Extract	IC_50_ Value (µg/mL) ^a^
*Lonicera japonica* Thunb.	7.92 ± 0.72
*Lysimachia christinae* Hance	13.19 ± 0.28
*Scirpus yagara* Ohwi	11.74 ± 0.77
*Paeonia* *suffruticosa* Andrew	7.69 ± 0.67
*Ailanthus altissima* (Mill.) Swingle	12.64 ± 0.37
*Pueraria thunbergiana* Benth	10.65 ± 0.04
*Curcuma zedoaria* (Christm.) Roscoe	10.13 ± 0.14
l-Penicillamine ^b^	8.24 ± 0.24

^a^ 50% inhibition concentrations (IC_50_, µg/mL) was calculated from the log dose inhibition curve and are expressed as mean ± S.E.M. of triplicate experiments. ^b^ Positive control.

**Table 3:** ONOO^−^ scavenging activity of subfractions from the 70% EtOH extract of root bark of *P. suffruticosa.*

Sample	IC_50_ Values (µg/mL) ^a^
70% EtOH extract	4.78 ± 0.13
CH_2_Cl_2_ fraction	1.78 ± 0.05
EtOAc fraction	0.25 ± 0.03
*n*-BuOH fraction	4.19 ± 0.23
H_2_O fraction	7.99 ± 0.79
l-Penicillamine ^b^	9.00 ± 0.38

^a^ 50% inhibition concentrations (IC_50_, µg/mL) was calculated from the log dose inhibition curve and are expressed as mean ± S.E.M. of triplicate experiments. ^b^ Positive control.

**Table 4:** Retention time, calibration equation, and correlation coefficient of methyl gallate.

Compound	Retention Time (min)	Calibration Equation ^a^	Correlation Coefficient (r^2^)
Methyl gallate	15.451	y = 17.430x + 4.221	0.996

^a^ y = peak area, x = concentration of standard (mg/mL).

**Table 5:** Contents of methyl gallate in the 70% EtOH extract and EtOAc fraction from root bark of *P. suffruticosa*.

Sample	Content (mg/g)	RSD ^a^ (%)
70% EtOH extract	5.07 ± 0.06	2.22
EtOAc fraction	39.62 ± 1.04	4.53

^a^ RSD = relative standard deviation.

**Table 6:** ONOO^−^ scavenging activity of methyl gallate.

Sample	IC_50_ Values (µM) ^a^
Methyl gallate	0.91 ± 0.26
l-Penicillamine ^b^	8.79 ± 0.17

^a^ 50% inhibition concentrations (IC_50_, μM) was calculated from the log dose inhibition curve and are expressed as mean ± S.E.M. of triplicate experiments. ^b^ Positive control.
